# Pan-histone deacetylase inhibitors regulate signaling pathways involved in proliferative and pro-inflammatory mechanisms in H9c2 cells

**DOI:** 10.1186/1471-2164-13-709

**Published:** 2012-12-18

**Authors:** Gipsy Majumdar, Piyatilake Adris, Neha Bhargava, Hao Chen, Rajendra Raghow

**Affiliations:** 1 Department of Veterans Affairs Medical Center, 1030 Jefferson Avenue, Memphis, TN, 38104, USA; 2 Department of Pharmacology, University of Tennessee Health Science Center, Memphis, TN, 38163, USA

**Keywords:** HDAC-inhibitor, Inflammation, Cardiac gene expression

## Abstract

**Background:**

We have shown previously that pan-HDAC inhibitors (HDACIs) m-carboxycinnamic acid bis-hydroxamide (CBHA) and trichostatin A (TSA) attenuated cardiac hypertrophy in BALB/c mice by inducing hyper-acetylation of cardiac chromatin that was accompanied by suppression of pro-inflammatory gene networks. However, it was not feasible to determine the precise contribution of the myocytes- and non-myocytes to HDACI-induced gene expression in the intact heart. Therefore, the current study was undertaken with a primary goal of elucidating temporal changes in the transcriptomes of cardiac myocytes exposed to CBHA and TSA.

**Results:**

We incubated H9c2 cardiac myocytes in growth medium containing either of the two HDACIs for 6h and 24h and analyzed changes in gene expression using Illumina microarrays. H9c2 cells exposed to TSA for 6h and 24h led to differential expression of 468 and 231 genes, respectively. In contrast, cardiac myocytes incubated with CBHA for 6h and 24h elicited differential expression of 768 and 999 genes, respectively. We analyzed CBHA- and TSA-induced differentially expressed genes by Ingenuity Pathway (IPA), Kyoto Encyclopedia of Genes and Genomes (KEGG) and Core_TF programs and discovered that CBHA and TSA impinged on several common gene networks. Thus, both HDACIs induced a repertoire of signaling kinases (PTEN-PI3K-AKT and MAPK) and transcription factors (Myc, p53, NFkB and HNF4A) representing canonical TGFβ, TNF-α, IFNγ and IL-6 specific networks. An overrepresentation of E2F, AP2, EGR1 and SP1 specific motifs was also found in the promoters of the differentially expressed genes. Apparently, TSA elicited predominantly TGFβ- and TNF-α-intensive gene networks regardless of the duration of treatment. In contrast, CBHA elicited TNF-α and IFNγ specific networks at 6 h, followed by elicitation of IL-6 and IFNγ-centered gene networks at 24h.

**Conclusions:**

Our data show that both CBHA and TSA induced similar, but not identical, time-dependent, gene networks in H9c2 cardiac myocytes. Initially, both HDACIs impinged on numerous genes associated with adipokine signaling, intracellular metabolism and energetics, and cell cycle. A continued exposure to either CBHA or TSA led to the emergence of a number of apoptosis- and inflammation-specific gene networks that were apparently suppressed by both HDACIs. Based on these data we posit that the anti-inflammatory and anti-proliferative actions of HDACIs are myocyte-intrinsic. These findings advance our understanding of the mechanisms of actions of HDACIs on cardiac myocytes and reveal potential signaling pathways that may be targeted therapeutically.

## Background

Regulation of gene expression is obligatorily dependent on the structure of chromatin that is dynamically remodeled via posttranslational modifications (e.g., acetylation, methylation, phosphorylation and ubiquitination) of its histone and non-histone constituents [1]. Reversible lysine acetylation represents a common modification of proteins that is carried out by histone acetyl transferases (HATs) and histone deacetylases (HDACs). The acetylation of histones leads to de-condensation of chromatin that becomes accessible to transcriptional machinery; in contrast, the inert chromatin is enriched in deacetylated histones [2,3]. Consistent with chromatin structure-dependent activation of gene expression, many transcriptional co-activators (e.g., GCN5, PCAF, CBP/p300) possess HAT activity whereas transcriptional co-repressors are associated with HDACs. Since DNA-binding domains are invariably missing from HATs and HDACs, they are recruited to their target promoters and enhancers via protein-protein interactions [2,3].

The HDACs represent an ancient super-family of enzymes conserved from yeast to man. The mammalian HDACs are divided into the “classical family” of 11 zinc-dependent hydrolases and the “non-classical family” of seven NAD^+^-dependent HDACs called sirtuins. Based on their phylogeny, domain organization and sub-cellular localization, the mammalian HDACs are further split into four sub-classes [4,5]. The HDAC members of class I (HDAC1, HDAC2, HDAC3 and HDAC8) contain a central deacetylase domain surrounded by short NH_2_ and COOH termini. Class I HDACs are mainly localized in the nucleus and possess potent enzymatic activity toward histones. Six members of Class II are further sub-grouped into Class IIa (HDAC4, HDAC5, HDAC7 and HDAC9) and Class IIb (HDAC6 and HDAC10), based on whether they possess one or two catalytic sites, respectively [4,5]. The class IV consists of a solitary member HDAC11, with homologies to Rpd3 and Hda1 proteins of yeast. Finally, sirtuins, the NAD-dependent lysine deacetylases, belong to Class III [6].

The actions of HATs and HDACs are intimately involved in the mechanisms of cardiac and skeletal muscle gene expression [7-10]. A number of studies have demonstrated a positive therapeutic potential of HDACIs in animal models of cardiac hypertrophy. The pan-HDACIs are thought to attenuate pathological cardiac hypertrophy via their ability to alter chromatin structure and gene expression in the heart, and in primary cultures of cardiac myocytes [8,11,12]. It is believed that by perturbing the epigenetic landscape of chromatin, the pan-HDAC inhibitors exert genome-wide changes in both myocytes as well as other cell lineages in the intact heart. However, the molecular underpinning of the altered gene expression in myocytes *versus* non-myocyte cells in the intact heart treated with pan-HDACIs is poorly understood. The batch-to-batch variability that is encountered in cardiac myocytes in primary cultures makes them less suitable to answer this question with rigor.

The H9c2 cells have emerged as an excellent *in vitro* alternative to primary cardiac myocytes. Although lacking the elaborate contractile apparatus of *bona fide* cardiac myocytes, H9c2 cells elicit robust hypertrophy-associated signature of fetal gene expression in response to angiotensin II, phenylephrine and IL-18; additionally, akin to what occurs in the intact heart, pathological hypertrophy of H9c2 cardiac myocytes could be attenuated by pan-HDAC inhibitors, TSA and CBHA [13-16]. This study was undertaken with an objective to determine genome-wide responses of H9c2 cardiac myocytes to two distinct pan-HDACIs. We exposed H9c2 cells to either CBHA or TSA for 6 and 24 h and analyzed their transcriptomes by whole-genome Illumina microarrays. We also subjected the differentially expressed genes of H9c2 cells, induced by CBHA and TSA treatments, to theoretical analyses using Ingenuity Pathway Analysis (IPA), Kyoto Encyclopedia of Genes and Genomes (KEGG) and Core_TF software programs. Our data revealed that although CBHA and TSA elicited unique signatures of gene expression at 6h and 24h time points, both HDACIs suppressed a number of common gene networks putatively involved in pro-inflammatory causes and consequences of pathological cardiac hypertrophy.

## Results

### H9c2 cardiac myocytes constitutively express all major HDACs and sirtuins

**Figure 1:**
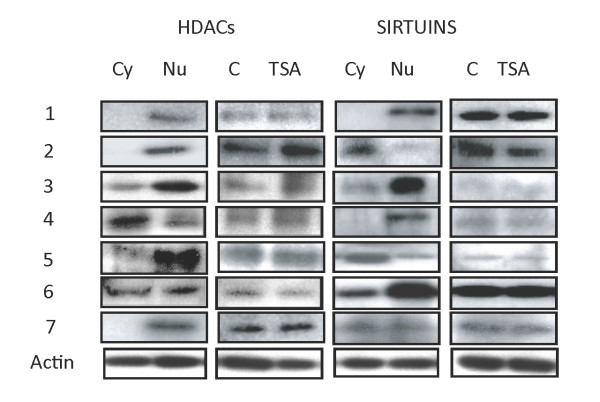
**Most HDACs and sirtuins are constitutively expressed in H9c2 rat cardiac myocytes and TSA treatment does not affect their expression.** Total cell extracts from control (C) and trichostatin treated cells (TSA) were probed with monospecific antibodies to the various enzymes as noted. Nuclear (Nu) and cytoplasmic (Cy) distribution of HDACs and sirtuins were similarly probed. The data are shown for untreated cells only. TSA-treatment did not change the subcellular distributions (Data not shown).

We have shown previously that IL-18-induced pathological hypertrophy in the intact heart and in H9c2 cells were attenuated by TSA and CBHA that caused hyper-acetylation of histones in the chromatin both *in vivo* and *in vitro*[14,17]. Modification of histones by pan-HDAC inhibitors are mediated by their ability to inhibit Class I and II HDACs; pan-HDAC inhibitors do not affect sirtuins [4,5,18]. Since the status of expression of various HDACs in H9c2 cells in not known, we began these studies by assessing the expression and sub-cellular localization of various HDACs and sirtuins in H9c2 cells. As shown in the representative western blots (Figure 1), although mono-specific antibodies readily detected all major HDACs and sirtuins their relative expression and subcellular localizations in the extracts of H9c2 cells were quite different. For example, HDAC-1, HDAC-2, HDAC-3, HDAC-5 and HDAC-7 are mainly localized in the nucleus of H9c2 cells that elicit nearly equal expression of HDAC-4 and HDAC-6 in their cytoplasm and nuclei. Evidently, whereas sirtuin-1, sirtuin-3, sirtuin-4 and sirtuin-6 are primarily localized in the nucleus, sirtuin-2 and sirtuin-5 are seen mainly in the cytoplasm. Finally, sirtuin-7 seems to be equally distributed in both cellular compartments (Figure 1). These data suggest that the subcellular compartmentalization of HDACs and sirtuins in the H9c2 cardiac myocytes is similar to that found in many other cells [4,5,18].

We also quantified steady state levels of cognate mRNAs of various HDACs and situins in H9c2 cells by qPCR. As shown in Table 1, H9c2 cells expressed HDAC-1 and HDAC-2 specific mRNAs most abundantly, followed by transcripts encoding HDAC-3>HDAC-7>HDAC-6>HDAC-5. Similar qPCR analyses revealed that the constitutive expression of sirtuin-2 specific mRNA was the highest in H9c2 cells that also expressed sirtuin-5> sirtuin-6> sirtuin-7> sirtuin-3>sirtuin-1>sirtuin-4 specific mRNAs (Table 1). Based on these and additional quantifications we surmised that there was a close correspondence between HDAC and sirtuin proteins and their cognate mRNAs. Additionally, we observed that exposure of H9c2 cells to the either pan-HDAC inhibitor affected neither the expression (Figure 1) nor sub-cellular distribution of HDACs or sirtuins (Data not shown).

**Table 1:** Quantitative PCR measurements of the steady state levels of various HDACs and sirtuins in H9c2 cells *

**Enzyme**	**δCp ( Mean ±SD )**	**Relative expression**
HDAC 1	1.36 ± 0.06	3.9
HDAC 2	0.2 ± 0.004	26.4
HDAC 3	2.26 ± 0.25	2.3
HDAC 4	5.28 ± 0.24	1
HDAC 5	4.92 ± 0.95	1.1
HDAC 6	3.43 ± 0.13	1.5
HDAC 7	2.81 ± 0.28	1.9
Sirtuin 1	6.06 ± 0.38	1.4
Sirtuin 2	2.17 ± 0.38	3.9
Sirtuin 3	6 .00 ± 0.38	1.4
Sirtuin 4	8.43 ± 0.39	1
Sirtuin 5	3.35 ± 0.29	2.5
Sirtuin 6	5.07 ± 0.38	1.7
Sirtuin 7	5.75 ± 0.59	1.5

* qPCR was carried out on total RNA extracted from H9c2 cells grown in serum free or serum supplemented media. Expression levels were calculated from δCp values from 3 replicate determinations. Expression levels of various HDACs were compared to HDAC 4 fixed at 1: similarly fold-changes in the expression of sirtuins were established against sirtuin 4 whose expression was fixed at 1.

### Pan-HDAC inhibitors alter global gene expression profiles of H9c2 cells

**Figure 2:**
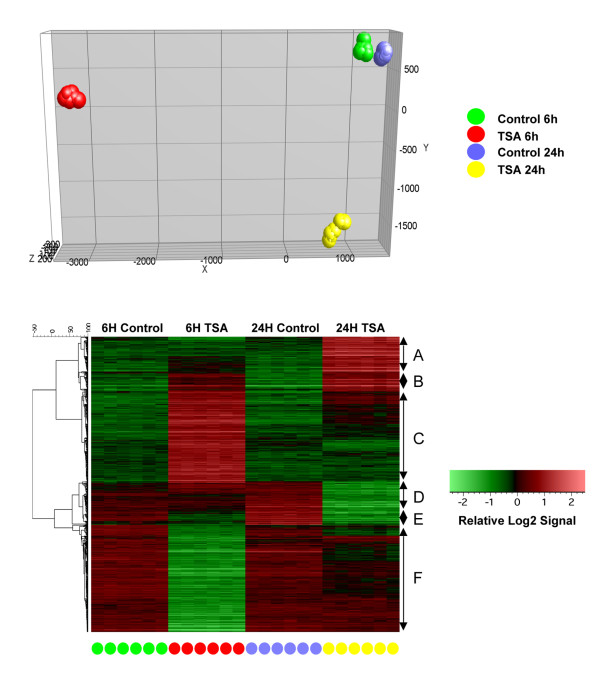
**PCA and clustered heat maps of DEGs in the H9c2 cells treated with TSA for 6 and 24 h.** PCA of gene expression dataset from six independent samples (denoted as colored spheres) of cardiac myocytes treated with TSA for 6 or 24 h shows unique locations of each treatment cohort (top panel). The transcriptomic data sets were filtered for significant differential expression based on Illumina detection values (> 0.99 for all samples for at least one group), fold change in treatment versus control group (>2.0 fold), ANOVA p-values (<0.05) and t-test p-values (<0.05) to identify DEGs that were arranged by unsupervised hierarchical clustering (bottom).

**Figure 3:**
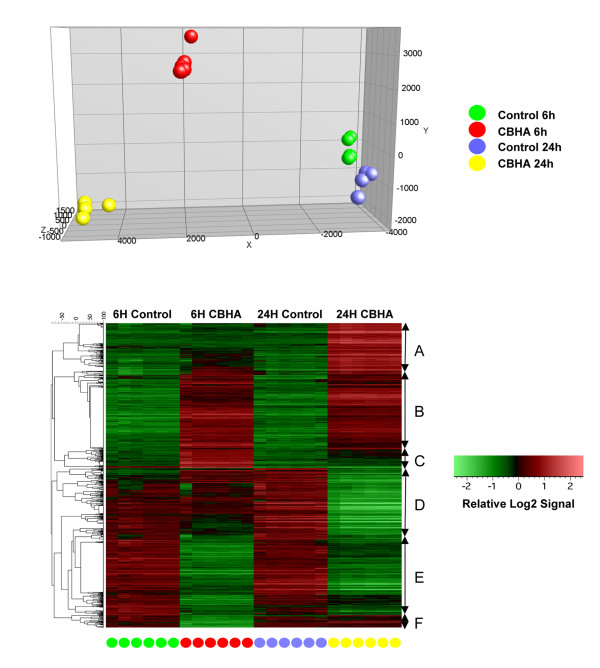
**PCA and clustered heat maps of DEGs in the H9c2 cells treated with CBHA for 6 and 24 h.** PCA of DEGs from six independent H9c2 cell samples representing CBHA treatment for 6 or 24 h shows unique locations of each treatment cohort (top panel). The heat maps of DEGs clustered using the statistical criteria described above and in Materials and Methods are shown (bottom).

The main aim of our study was to examine the effect of HDACIs on gene expression in cardiac myocytes without other cell types (e.g., endothelial cells and fibroblasts) that coexist in the intact heart. We serum-starved H9c2 cells for 16-24h before initiating drug treatment by incubating the cells in complete growth medium (control) and growth media supplemented with CBHA or TSA. Based on our empirical assessment of the actions of HDACIs in “cell cycle-synchronized” H9c2 cells, in the presence or absence of IL-18 [14], we believe that 6h and 24h time points of treatment will yield snapshots of genome-wide actions of CBHA and TSA during early and late stages of cell cycle. Messenger RNAs extracted from six replicates of each treatment cohort were processed for hybridization to Illumina rat microarrays and subsequent analysis. We filtered the gene expression dataset through the criteria of absolute 2-fold change and p value of <0.01 before analyzing these data by principal component analysis (PCA) and the un-supervised hierarchical clustering methods. As shown in Figures 2 and 3, the cohorts (6 replicates each) of vehicle-treated H9c2 cells harvested at 6h and 24h occupy close, albeit unique positions in the PCA graph. Similarly, the replicates of CBHA- or TSA-treated cells harvested at 6h and 24 h after treatment are also uniquely grouped in the PCA graph.

**Figure 4:**
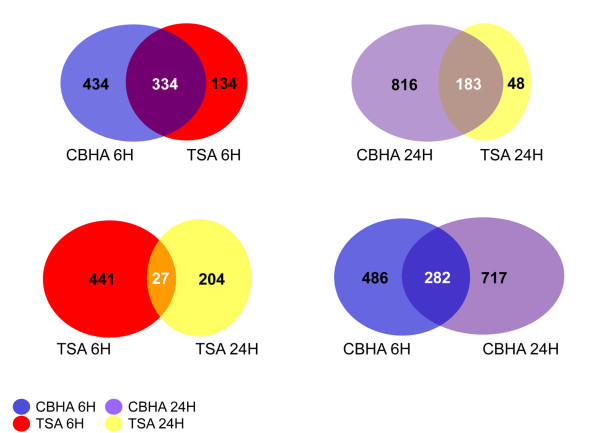
**Venn diagrams show the number of differentially expressed probe sets elicited in response to CBHA or TSA treatment of cardiac myocytes for 6 or 24 h.** The number of genes affected by CBHA is almost twice that elicited by TSA. There is lesser overlap among the DEGs elicited early (6 h) and late (24 h) for both TSA and CBHA compared with a much larger number of common transcripts induced by TSA and CBHA at either time point.

The RatRef-12 Expression BeadChip contains about 21,900 genes. Exposure of H9c2 cells to TSA and CBHA led to a total of 672 and 1485 differentially expressed genes, respectively. It appears therefore that the expression of approximately 3% and 6% of genes were significantly affected in H9c2 cells in response to TSA and CBHA, respectively. Based on their temporal expression characteristics and quantification of their expression levels, the TSA- and CBHA-responsive genes could be organized into six distinct clusters, A through F (Figures 2 and 3). The sizes of Clusters C and F elicited in TSA-treated cells were much larger compared with their counterpart clusters in CBHA-treated cells. This is in contrast to what occurred in H9c2 cells treated with CBHA that induced more numerous transcripts belonging to Clusters B, D and E. As depicted in Figure 4, TSA elicited differential expression of 468 and 231 genes at 6h and 24h post-treatment, respectively. An identical exposure of H9c2 cells to CBHA for 6h and 24h elicited 768 and 999 DEGs, respectively.

### Ingenuity pathway analysis indicates that CBHA and TSA perturb overlapping yet distinct gene networks in H9c2 cardiac myocytes

We began our gene network studies with the reasoning that interrogation of the maximum numbers of DEGs by IPA would reveal the most robust networks involved in the actions of TSA or CBHA. Therefore, at first, we merged all DEGs contained in Clusters A through F into a single dataset. However, we discovered that the combined dataset was too large for an optimal analysis by the IPA program and thus, with a goal to reduce the number of DEGs that could be assessed by IPA, we re-filtered the TSA- and CBHA-responsive DEGs through more stringent statistical criteria. We set an absolute 2.5-fold change and p value of <0.01 for TSA-responsive genes; similarly, CBHA-responsive genes were re-filtered through an absolute 3.5-fold change and a p value of <0.01. These statistical maneuvers reduced TSA-regulated genes to 157 and 114, at 6h and 24h post treatment. Of these, 52 genes were up regulated (33%) at 6h and 104 genes down regulated (66%). At 24h treatment 52 genes were up regulated (45%) and 62 genes were down regulated (66%). A more stringent statistical analysis yielded 147 and 249 genes for CBHA treatment at 6h and 24h, respectively. At 6h treatment of CBHA 82 genes were up regulated (56%) and 65 genes down-regulated (44%). At 24h treatment 90 genes were up regulated (36%) and 159 genes were down regulated (64%).

**Figure 5:**
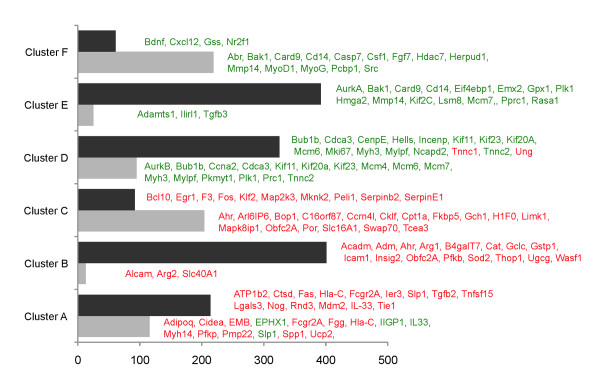
**H9c2 cardiac myocytes elicit unique gene expression in response to TSA or CBHA at 6h.** A vast majority of genes in clusters **A**, **B** and **C** was up regulated (red) by TSA and CBHA while the expression of genes in clusters **D**, **E**, and **F** was suppressed (green) by both HDACIs.

**Figure 6:**
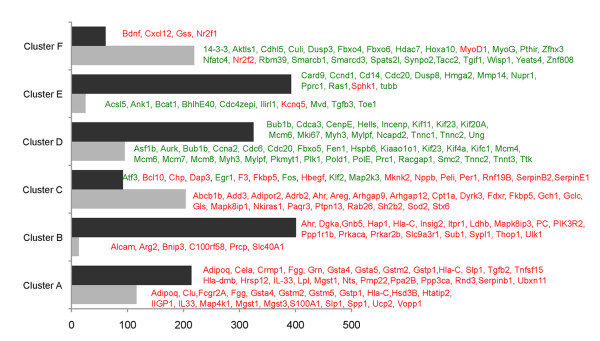
**Major genes regulated by TSA or CBHA after 24 h.** Incubation of H9c2 cells with either CBHA or TSA for 24 h led to enhanced (red) expression of most genes in clusters **A**, **B** and **C** while both HDACIs suppressed (green) a vast majority of the transcripts in clusters **D**, **E** and **F**.

The initial analysis of the merged datasets by IPA revealed that although CBHA and TSA elicited unique signatures of gene expression, the two pan-HDAC inhibitors also impinged on numerous common gene targets at 6h (334) and 24h (183) post-treatment (Figure 4). We also observed that genes in Clusters A through C were generally up regulated by both HDACIs; in contrast, expression of most of the mRNAs contained in Clusters D through F was repressed by both CBHA and TSA (Figures 5 and 6).

**Figure 7:**
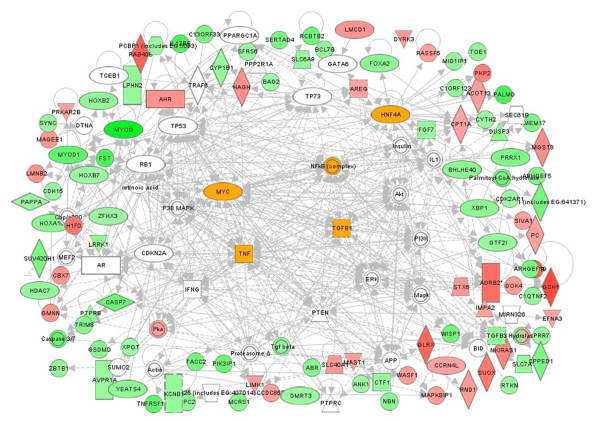
**Major gene networks specifically modulated by TSA at 6h.** The IPA merged the top seven networks of TSA-elicited genes into a single network. The dominant nodes of TGFβ, HNF-4A, TNFα and MYC connected extensively to the signaling node of NFkB are highlighted. The major gene networks are either directly (solid lines) or indirectly (broken lines) connected to PI3K- and MAPK-specific signaling pathways. The up regulated genes are shown in pink and red while down-regulated genes are depicted in green.

Next, we combined the top seven IPA networks of TSA-specific DEGs at 6h and 24h to reveal the hierarchy of the potential gene networks in the actions of the two pan-HDACIs (Tables 2 and 3). The DEGs seen after 6h treatment with TSA revealed the existence of TGFβ (33 connections; 16 focus genes) TNF-α (35 connections; 10 focus genes) and IFNγ (32 connections; 10 focus genes) specific gene networks (Table 2 and Figure 7). These cytokine hubs were connected with signaling kinases such as PTEN-PI3K-AKT (53 connections; 11 focus genes) and MAPK (49 connections with 11 focus genes), and transcription factors (HNF4A; 30 connections with 14 focus genes), (Myc; 30 connections with 12 focus genes) and (NFkB; 33 connections with 10 focus genes). We should note here that the inflammatory cytokine hubs are connected to genes that were either induced or suppressed by TSA (Figures 5 and 6). Thus, TNF-α specific hub was connected to HDAC-7, cardiotrophin, MyoD and Myogenin, all of which were down regulated; in contrast, the expression of geminin (GMNN) was induced by TSA (Figure 7). Similarly, the IFNγ specific hub is connected to both TSA-inducible (XPOT, caspase-3 and caspase-7) and TSA-suppressible (PKA) genes. Finally, PTEN specific hub is connected to two microtubule-associated kinases MAST1 and LIMK1 that were up regulated by TSA and a transcription factor (GTF21) that was down regulated in TSA-treated H9c2 cells post 6h treatment. These data are consistent with our earlier report showing that the expression of PTEN was highly induced by CBHA in H9c2 cells [14] and in response to both CBHA and TSA in the intact heart [17].

**Table 2:** Time-dependent elicitation of cytokine and signaling pathway by TSA in H9c2 cells*

**Control versus TSA (6 h)**		**Control versus TSA (24 h)**	
**Gene pathway**	**Total connections (No. of focus genes)**	**Gene pathway**	**Total connections (No. of focus genes)**
TGFβ1	33 (16)	TGFβ1	29 (18)
HNF4A	30 (14)	HNF4A	30 (12)
MyC	30 (12)	ERK-p38-JNK	56 (10)
PTEN-PI3K-Akt	53 (11)	TP53	33 (10)
TNF-α	35 (10)	TNF-.α	30 ( 8 )
NFκΒ	33 (10)	FOS	28 ( 8 )
p38-JNK	45 ( 9 )	CDKN1A	12 ( 8 )
IFNγ	32 ( 9 )	NFκΒ	29 ( 3 )
Caspase 3	28 ( 8 )	Calcium	28 ( 3 )
CDKN2A	10 ( 8 )	AP1	18 ( 3 )
Calcium	31 ( 6 )	CaMKII	10 ( 3 )
Insulin	22 ( 6 )	Caspase 3	16 ( 2 )
TP53	20 ( 5 )		

*DEGs in response to TSA treatment of H9c2 cells for 6 and 24 h were subject to IPA as detailed in “Material and Methods”. Gene pathways with total number of connections (direct and indirect) and focus genes are hierarchically arranged according to the number of focus genes. The relative order of the p53-specific network elicited at 6 h (13th) and 24 h (4th) is mechanistically significant.

**Table 3:** Key cytokine and signaling pathway elicited in response to CBHA at 6h and 24h in H9c2 cells*

**Control versus CBHA (6 h)**		**Control versus CBHA (24 h)**	
**Gene pathway**	**Total connections (No. of focus genes)**	**Gene pathway**	**Total connections (No. of focus genes)**
TNF	45 (22)	CDKN1A	65 (35)
Myc	43 (18)	TP53	60 (35)
HNF4A	32 (18)	IL-6	50 (34)
PTEN-PI3K-Akt	76 (17)	HNF4A	41 (30)
ERK-p38-JNK	73 (17)	CDKN2A	43 (25)
NFκΒ	40 (16)	ERK- p38-JNK	52 (21)
IFNγ	45 (12)	NFκΒ	41 (17)
FOS	43 ( 8 )	IFNγ	38 (17)
AP1	31 ( 8 )	Cyclin A	29 (12)
Insulin	28 ( 8 )	PI3K-Akt	41 (10)
TP73	16 ( 7 )	MAPK	24 ( 8 )
CDKN2A	20 ( 5 )	Insulin	24 ( 5 )

*DEGs in response to CBHA were subject to IPA as detailed in “Material and Methods”. Gene pathway with total number of connections (direct and indirect) and focus genes are hierarchically arranged according to the number of focus genes. The relative position of the p53-specific network elicited at 6 h (11th) and 24 h (2nd) is significant.

**Figure 8:**
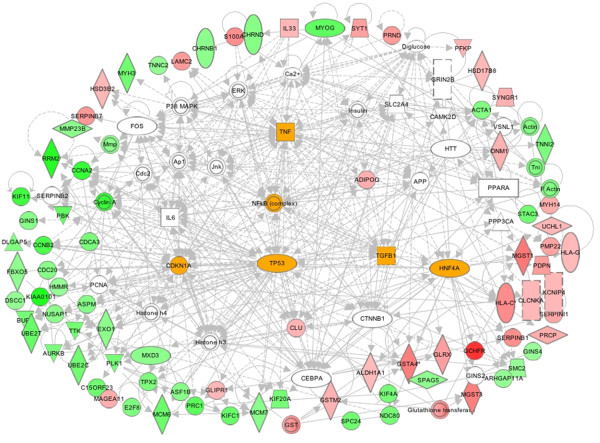
**Major intracellular networks of cardiac genes that were differentially modulated by TSA at 24h.** The dominant nodes, centered by TGFβ, TP53, HNF-4A.TNF.α, CDKN1A and NFkB, are highlighted; signaling nodes of ERK-JNK-p38MAPK are also present as prominent nodes.

A continued exposure to TSA for 24h led to apparent consolidation of the TGFβ (29 connections with 18 focus genes) and TNF-α (30 connections; 8 focus genes) specific gene networks (Table 2 and Figure 8). However, in contrast to a dominant involvement of PTEN-PI3K-AKT signaling seen at 6h (Table 2), at 24h, MAPK signaling (65 connections; 12 focus molecules) connected with TGFβ and TNF-α specific hubs was prominent. There were also unique signal transduction and transcription factor specific networks elicited by TSA at 24 h; thus in addition to HNF4A, TSA strongly induced Ap1-Jun/Fos (46 connections; 11 focus molecules), p53 (33 connections; 10 focus molecules) and cyclin-dependent kinases (Table 2 and Figure 8). At 24h treatment, TNF-αspecific gene networks were associated with regulators of cell cycle (Kif 20A, cyclin A), chromatin architecture and transcription (MyoG, MyoD, F-actin, ACTA-1, MCM7); TSA down regulated all these mRNAs. These gene network analyses are consistent with the hypothesis that TSA blunted the pro-inflammatory and pro-fibrotic actions of TNF-α and TGFβ.

**Figure 9:**
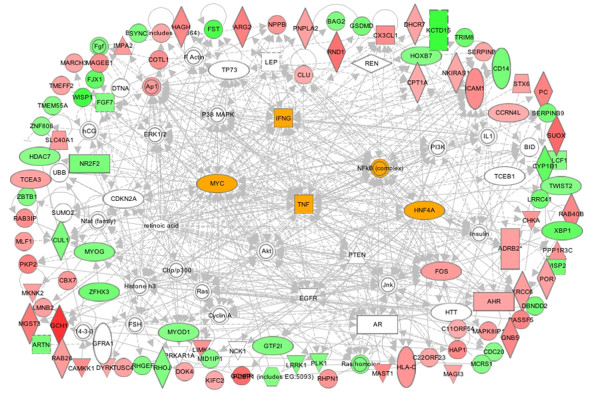
**The major gene networks specifically modulated by CBHA at 6h.** The IPA merged the top seven networks of CBHA-elicited genes into a single network. The dominant nodes of IFNγ, HNF-4A, TNFα and MYC connected extensively to the signaling node of NFkB are highlighted. The major gene networks are either directly (solid lines) or indirectly (broken lines) connected to PI3K- and MAPK-specific signaling pathways. The up regulated genes are shown in pink and red while down-regulated genes are depicted in green.

**Figure 10:**
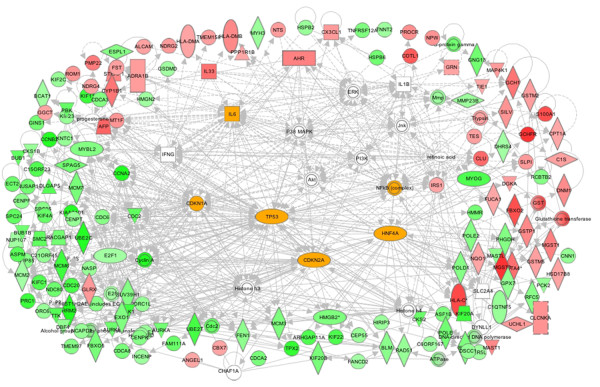
**Major intracellular networks of cardiac genes that were differentially modulated by CBHA at 24h.** The dominant nodes, centered by IL-6, TP53, CDKN1A, CDKN2A, HNF-4A and NFkB are highlighted; signaling nodes of ERK-JNK-p38MAPK are also present as prominent node.

Evidently the signaling and transcriptional regulatory gene networks elicited in CBHA-treated H9c2 cells for 6h or 24h also evolved with treatment duration (Table 3). The IPA of DEGs of cells treated for 6h with CBHA revealed the existence of TNF-α (45 connections; 22 focus genes) and IFNγ (45 connections; 12 focus genes) specific gene networks (Table 3 and Figure 9). These two cytokine hubs were connected with PTEN-PI3K-AKT (76 connections; 17 focus genes), MAPK (70 connections with 14 focus genes), and transcription factors (Myc; 43 connections with 18 focus genes; HNF4A; 32 connections with 18 focus genes and NFkB; 40 connections with 16 focus genes). We should note however, that although PTEN-PI3K-AKT and MAPK signaling molecules were robustly elicited by both CBHA and TSA, the cytokine specific networks induced by the two HDACIs were significantly different in detail. For example, while TSA preferentially elicited TGFβ-intensive gene networks both at 6h and 24h, CBHA treatment elicited strong TNF-α and IFNγ specific networks at 6h whereas cells exposed for 24h induced IL-6 (50 connections; 34 focus genes) and IFNγ-centered (38 connections; 17 focus genes) hubs. Strong CDKN-specific (137 connections; 72 focus genes) and p53-specific (60 connections; 35 focus genes) gene networks (Table 3 and Figure 10) were also seen in CBHA-treated cells at 24h.

A number of unique and shared features of the two pan-HDACIs are worth mentioning here. First, the TNF-α specific networks seen in CBHA-treated cells at 6h were similar to those seen in TSA-treated cells; in both cases TNF-α specific hubs were directly connected with MyoD, MyoG, HDAC 7, SERPINB9 genes, all of which were down regulated (Figure 9). Second, the PTEN specific gene network, connected to genes that were either induced (MAST1) or suppressed (PLK1, CDC20 and GTF21) by CBHA, was only seen at 6h after CBHA treatment. Third, the TP53 gene network was more prominent in CHBA-treated cells at 24h (60 connections; 35 focus genes) compared with that seen in TSA-treated cells after 24h. Fourth, numerous DEGs involved in the regulation of cell cycle, chromatin remodeling and mRNA metabolism were affected by TSA and CBHA. Finally, it is significant to note that the pro-inflammatory IFNγ and IL-6 specific gene networks were connected mainly to down regulated genes involved in DNA replication cell cycle cell cycle (MCM7, CENT, MCM6, CCNA2, CDC2, CDC6, Cycin A, E2F1) in CBHA-treated cells at 24h.

### Ingenuity pathway analyses of six unique clusters of DEGs corroborate and extend the TSA- and CBHA-inducible gene networks seen in the combined dataset

As outlined above, the merged dataset was devoid of a large number of DEGs that were contained in Clusters A through F. Therefore, to carry out a more comprehensive network analysis with a goal to corroborate and extend IPA of the merged dataset, we analyzed Clusters A through F (elicited at 6h and 24h by CBHA and TSA) individually (Additional files 1, 2, 3, 4: Figures S1-4). These analyses revealed that, irrespective of the HDACI or the duration of the treatment, Clusters A, B and C were populated by genes that regulate intracellular signaling, cellular energetics, inflammation and proliferation and apoptosis. The TSA-responsive Clusters A-C at 6h (Additional file 1: Figure S1) or 24 h (Additional file 2: Figure S2) elicited prominent TNF-α, HNF-4A, IFNγ YY1, Egr1, E2F, and TP53 specific nodes that are connected to gene networks involved in metabolic regulation, cellular energetics and proliferation and apoptosis (SLP1, SPP1, and DDX5). CBHA-responsive Clusters A-C at 6h (Additional file 3: Figure S3) or 24 h (Additional file 4: Figure S4) elicited TNF-α IFNγ, NFκB, YY1, E2F and TP53 connected with molecules known to regulate immunity, inflammation, intermediary metabolism, and cell growth (PRKACA, PIK3R2, GNB5, MAPK8IP1, ADIPOR2 and EGR1). Only Clusters depicting strong networks are shown. Majority of the genes in Clusters A-C are up regulated by TSA or CBHA irrespective of the duration of treatment (Figures 5 and 6).

The genes in Clusters D, E and F were repressed by both pan-HDACIs, regardless of the duration of treatment (Figure 5 and 6). As compared to TSA, CBHA elicited a much larger Cluster D in H9c2 cells (325 *versus* 95 genes). Cluster D was populated by genes known to control organization and replication of DNA (e.g., POLD1, SMC2 and MCM6), cell cycle (e.g., CCNA, CCND, BUB1b, CENPE and CDC6) and skeletal muscle structure (e.g., MYH3, TNNT, TNNC2). Regardless of the duration of treatment, both CBHA- and TSA-responsive Cluster D genes formed strong p53, YY1 and Cyclin-CDK specific networks (Additional files 1, 2, 3, 4; Figures S1-S4). The regulators of nuclear organization (e.g., NUPR1, HMGA2, and TUBB), cell cycle (e.g., CCND1, GTSE and CDC20) and apoptosis (e.g., CARD9 and CASP7) dominated Clusters E and F of cells treated with either pan-HDAC inhibitor, irrespective of the duration of treatment. However, the strongest networks in TSA-responsive genes were demonstrated in Clusters F involving TNF-α, IL-6 and IFN-γ at 6 and 24h (Additional files 1 and 2; Figures S1 and S2). The CBHA-responsive genes demonstrated strong networks in Clusters E and formed TNF-α-, IFN-γ-, TP53- and cyclins/CDK specific gene networks at 6 and 24 h (Additional files 3 and 4: Figures S3 and S4). We may sum up the results of IPA of Clusters A through F individually by concluding that these analyses not only validated the prediction of IPA of the combined dataset, but also unraveled the existence of additional gene networks. Thus, in addition to the existence of gene networks representing cytokines (TGFβ1, TNF-α and IFNγ), signal transduction pathways (PTEN-PI3K-AKT and MAPK) and transcription factors (p53, NFκB and HNF4A), the IPA of the DEGs in the Clusters A through F unraveled the putative involvement of Egr1, YY1, E2F, and STAT3 specific gene networks in the actions of the two pan-HDAC inhibitors.

### KEGG analysis of differentially expressed genes induced by CBHA and TSA

To extend the *in silico* examination of the differentially regulated genes by IPA, we subjected DEGs that were common to TSA and CBHA to KEGG analysis. The KEGG program is designed to convert the molecular interactions and gene networks into biologically functional pathways (Table 4). The KEGG analysis revealed that CBHA and TSA elicited a number of overlapping pathways, regardless of the duration of the treatment (Table 4). Thus, phosphatidylinositol metabolism and signaling and MAPK pathways were preeminent in H9c2 cells exposed to either TSA or CBHA at 6h. Furthermore, the putative PTEN-PI3K-AKT/PKB signaling pathways were connected with numerous genes involved in the metabolism of pyruvate, citrate and amino acids, as well as in the intermediary metabolism of purines and pyrimidines (Table 4). The emergence of gene networks known to regulate cell cycle and DNA replication, metabolism of xenobiotics, oxidative stress and extracellular matrix were also common in H9c2 cells incubated with either CBHA or TSA for 24h (Table 4). Based on these observations we surmise that similar HDACI-induced gene networks were uncovered by IPA and KEGG analyses.

**Table 4:** KEGG pathways represented by DEGs commonly regulated by TSA and CBHA at 6 h or 24 h in H9c2 cells*

**DEGs common to TSA and CBHA at 6h**			
**KEGG pathway**	**Gene nos**	**Entrez Gene IDs**	**P value**
MAPK signaling pathway	7	FGF7, FGF8, MKNK2, NFAT4,TGFb3,	P=2.02e-3
		CD14, MAPKI8P1	
Adipocytokine signaling pathway	5	CPT1A, PEPCK, ACSL5, ADIPOR2, CAMKK1,	P=9.51e-5
Purine metabolism	5	NME6, GMPR, IMPDH2, POLD3, POLR3F	P=2.90e-3
Cell cycle	5	TGFb3, CUL1, CCNA2, CCNB2, CDC20	P=8.67e-4
Phosphatidylinositol signaling system	4	IMPA2, INPP4A, PIK3C2A, ITPKA	P=1.14e-3
Ubiquitin mediated proteolysis	3	UBE2C, CUL1, CDC20	P=2.88e-3
TGF-beta signaling pathway	3	FST, TGFb3, CUL1	P=1.60e-2
PPAR signaling pathway	3	CPT1A, PEPCK, ACSL5	P=1.60e-6
Pyruvate metabolism	3	HAGH, PCB, PEPCK	P=1.60e-7
Inositol phosphate metabolism	3	IMPA2, INPP4A ITPKA	P=1.60e-8
Tryptophan metabolism	3	CYP1B1, MAOA, LCMT1	P=1.60e-9
Pyrimidine metabolism	3	NME6 ,POLD3, POLR2D	P=1.60e-10
Citrate cycle (TCA cycle)	2	PC, PEPCK	P=1.60e-11
Fatty acid metabolism	2	CPT1A, ACSL5,	P=1.60e-12
Glutamate metabolism	2	GCLC, GLS	P=1.60e-13
Arginine and proline metabolism	2	ARG2, MAOA	P=1.60e-14
Histidine metabolism	2	MAOA, LCMT1	P=1.60e-15
Tyrosine metabolism	2	MAOA, LCMT1	P=1.60e-16
Selenoamino acid metabolism	2	SEPHS2, LCMT1	P=1.60e-17
Glutathione metabolism	2	GCLC, MGST3	P=1.60e-18
**DEGs common to TSA and CBHA at 24h**			
Cell cycle	12	MCM4, MCM3, MCM6 MCM7, PLK1, BUB1B,	P=1.74e-14
		BUB1 CCNA2, CCNB2 PKMYT1 CDC6, CDC20	
Glutathione metabolism	6	GPX7, GST4, GSTM1, GSTP1, GCLC, MGST3	P=1.15e-8
Metabolism of xenobiotics by cytochrome P450	5	MGST1, GST4, GSTM1, GSTP1, MGST3	P=2.76e-6
Pyrimidine metabolism	4	POLD1, POLE2, RFC5, RRM2	P=3.95e-4
Purine metabolism	4	POLD1, POLE2, RFC5, RRM2	P=2.48e-3
DNA polymerase	3	POLD1, POLE2, RFC5	P=1.20e-4
ECM-receptor interaction	2	HMMR, LAMC2	P=3.99e-2
Ubiquitin mediated proteolysis	2	UBE2C, CDC20	P=1.19e-2

*The KEGG pathways associated with the common gene set affected by TSA or CBHA at 6h and 24h post-treatment in H9c2 cells are hierarchically arranged based on the number of common DEGs and the significance of enrichment (p value) derived as described in “Material and Methods”.

### A putative involvement of MAPK pathways in the action of pan-HDAC inhibitors

**Figure 11:**
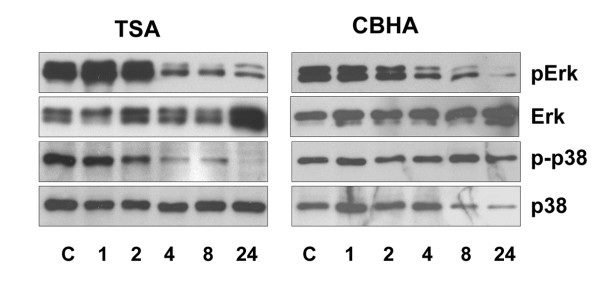
**HDAC inhibitors affect phosphorylation of ERK and p38 in H9c2 cells.** Cells were incubated with or without TSA or CBHA at different time intervals as shown. Ten microgram aliquots of protein extracts were separated by 10% SDS-PAGE. Western blots were probed with mono-specific antibodies against phospho-ERK or p38 MAPK. Blots were stripped, washed and re-probed with anti-ERK and p38 antibodies. Representative western blots of ERK and p38 MAPK in response to TSA and CBHA are shown.

The network analyses of genes that were differentially regulated by CBHA and TSA, regardless of whether it was done by IPA or KEGG programs, strongly predicted a role of PTEN-PI3K-AKT/PKB and MAPK signaling pathways in the actions of HDACIs. We reported earlier that both CBHA and TSA potently induced the expression of PTEN and concomitant reduction in PI3K and AKT phosphorylation in H9c2 cells as well as in the intact heart [17]. To test a potential role of MAP kinases*,* we extracted proteins from H9c2 cells incubated with CBHA or TSA for various time intervals and assessed the steady state levels of total and phosphorylated ERK, JNK and p38 MAPK. As shown in Figure 11, an exposure to TSA for 4h led to a reduced phosphorylation of ERK (pERK) and its phosphorylation remained inhibited until 24h. TSA treatment also significantly suppressed phosphorylation of p38 (p-p38) as early as 2h. Finally, an exposure of H9c2 cells to CBHA resulted in a reduction of pERK at 4h, while the levels of p-p38 kinase were not significantly affected by CBHA. The temporal changes in the regulation of JNK in response to CBHA or TSA were inconclusive (Data not shown). Finally, it should be noted that neither TSA nor CBHA altered the steady state levels of total ERK or p38 kinases (Figure 11).

### Frequency of putative transcription factor binding sites in differentially expressed genes in response to CBHA and TSA

With an aim to elucidate potentially common pathways involved in the induction of genes by CBHA and TSA, we extended gene network analyses by an *in silico* examination of transcription factor binding sites (TFBS) in the promoters of DEGs. We explored 1 kb of DNA upstream of transcription start site of all differentially expressed genes by CORE_TF, a web-based program that identifies dominant TFBS. As shown in Table 5, in DEGs induced by CBHA at 6 and 24h, the topmost transcriptional factor motifs were those of AP2, CHCH, E2F1, EGR2 and ETF. An over-representation of AP2, CHCH, E2F1, EGR2 and ETF was also seen in TSA-treated cells; additionally, the promoters of the TSA-induced DEGs expressed zinc finger-containing transcription factors (Sp1 and KROX). Finally, NF-Y specific motifs were overrepresented in DEGs induced by TSA at 24h. The preponderance of E2F1, EGR2, Sp1 and KROX transcription factor binding sites in the DEGs induced by either pan-HDAC inhibitor was consistent with an ability of these transcription factors to regulate genes involved in cell proliferation and apoptosis. The members of the E2F family, that bind to RB1, also play a key role in regulating G to S transition; similarly, NF-Y has a fundamental role in the expression of genes that regulate G2/M phase of the cell cycle.

**Table 5:** Enriched transcription factor binding sites in the promoters of genes, obtained from H9c2 cells treated with CBHA or TSA*

**CBHA 6h**				
**Transcription factors**	**# exp promoters hit**	**# exp promoters**	**P value**	**Q value**
AP2_Q6	584	658	0	0
CHCH_01	617	658	0	0
E2F_Q2	548	658	0	0
E2F1_Q3_01	317	658	0	0
EGR2_01	592	658	0	0
ETF_Q6	412	658	0	0
GC_01	584	658	0	0
KROX_Q6	411	658	0	0
LRF_Q2	456	658	0	0
MAZR_01	233	658	0	0
NGFIC_01	434	658	0	0
SP1_Q6_01	558	658	0	0
WT1_Q6	528	658	0	0
AHRHIF_Q6	361	658	1.00E-09	2.25E-08
MUSCLE_INI_B	593	658	1.93E-08	3.97E-07
AHRARNT_02	336	658	1.54E-07	3.03E-06
SPZ1_01	605	658	1.77E-07	3.35E-06
AP2ALPHA_01	596	658	3.81E-07	6.21E-06
AP2GAMMA_01	622	658	3.63E-07	6.21E-06
MAZ_Q6	315	658	3.70E-07	6.21E-06
UF1H3BETA_Q6	31	658	1.20E-05	1.89E-04
CACBINDINGPROTEIN_Q6	431	658	1.26E-05	1.92E-04
MOVOB_01	647	658	3.16E-05	4.44E-04
VDR_Q3	603	658	3.42E-05	4.62E-04
ZF5_B	655	658	9.53E-05	1.25E-03
E2F1DP2_01	293	658	1.48E-04	1.89E-03
NFY_Q6_01	226	658	2.11E-04	2.62E-03
CACD_01	635	658	2.61E-04	3.16E-03
MZF1_02	571	658	3.58E-04	4.23E-03
CACCCBINDINGFACTOR_Q6	78	658	4.56E-04	5.26E-03
WHN_B	145	658	4.92E-04	5.54E-03
**CBHA 24h**				
AP2_Q6	728	847	0	0
AP2ALPHA_01	776	847	0	0
CHCH_01	794	847	0	0
E2F_Q2	680	847	0	0
E2F1_Q3_01	379	847	0	0
EGR_Q6	604	847	0	0
ETF_Q6	492	847	0	0
GC_01	737	847	0	0
KROX_Q6	486	847	0	0
MAZR_01	256	847	0	0
NGFIC_01	532	847	0	0
SP1_01	697	847	0	0
WT1_Q6	682	847	0	0
LRF_Q2	559	847	2.00E-10	4.68E-09
MZF1_02	759	847	2.00E-10	4.68E-09
SPZ1_01	780	847	1.80E-09	4.02E-08
AP2GAMMA_01	802	847	3.60E-09	7.33E-08
MOVOB_01	837	847	1.79E-08	3.49E-07
CACBINDINGPROTEIN_Q6	555	847	8.55E-07	1.48E-05
CACD_01	822	847	1.19E-06	1.99E-05
CACCCBINDINGFACTOR_Q6	108	847	2.31E-06	3.72E-05
AHRHIF_Q6	431	847	3.66E-06	5.53E-05
NFY_Q6_01	294	847	1.24E-05	1.77E-04
E2F1DP2_01	378	847	1.64E-05	2.26E-04
AHRARNT_02	408	847	1.99E-05	2.67E-04
MAZ_Q6	382	847	3.46E-05	4.51E-04
STRA13_01	32	847	8.99E-05	1.08E-03
SP3_Q3	821	847	3.89E-04	4.14E-03
VDR_Q3	766	847	4.08E-04	4.24E-03
MUSCLE_INI_B	733	847	7.95E-04	8.10E-03
**TSA 6h**				
E2F_Q2	319	390	0	0
E2F1_Q3_01	187	390	0	0
ETF_Q6	243	390	0	0
GC_01	341	390	0	0
KROX_Q6	233	390	0	0
SP1_Q6_01	320	390	0	0
CHCH_01	361	390	8.00E-10	3.33E-08
MAZR_01	128	390	1.70E-09	6.52E-08
EGR2_01	350	390	1.66E-08	5.52E-07
WT1_Q6	309	390	6.34E-08	1.98E-06
NGFIC_01	249	390	8.85E-08	2.60E-06
AP2_Q6	334	390	7.62E-07	2.00E-05
LRF_Q2	262	390	8.74E-07	2.18E-05
UF1H3BETA_Q6	20	390	8.70E-05	2.07E-03
AHRHIF_Q6	205	390	9.40E-05	2.10E-03
AHRARNT_02	195	390	1.99E-04	4.14E-03
MAZ_Q6	183	390	2.74E-04	5.48E-03
ZEC_01	2	390	3.48E-04	6.69E-03
**TSA 24h**				
GC_01	161	194	1.45E-05	2.49E-03
NFY_01	118	194	9.66E-06	2.49E-03
E2F_Q2	141	194	4.78E-05	4.94E-03
SP1_Q2_01	143	194	8.48E-05	7.30E-03

* Overrepresented transcription factor binding sites were found in genes obtained from H9C2 cells induced with CBHA and TSA at different time points using CORE_TF. Differential expression of genes was considered to obtain the genes with 2 fold change.

* Experimental promoters hit shows the number of genes which have that TFBS in their promoter region, from the total number of genes scanned.

* One-tailed Mann–Whitney rank sum p value.

* Multiple testing corrected false discovery rate (FDR) q value, here q value <0.01.

## Discussion

We report here a comprehensive analysis of gene networks in H9c2 cells induced in response to two distinct pan-HDAC inhibitors, TSA and CBHA that have been shown to attenuate cardiac hypertrophy *in vivo* and *in vitro*[14,17]. Although H9c2 cells differ from *bona fide* cardiac myocytes in their inability to elicit well-defined sarcomeres, they elicit a pathological hypertrophy-specific gene expression program in response to Angiotensis II, IL-18 and phenylephrine. Furthermore, pan-HDAC inhibitors alleviated the hypertrophy response of H9c2 cells as judged by their molecular phenotype [13-15]. We show that both pan-HDACIs induced intracellular energetics and pro-inflammatory cytokine specific gene networks that were connected with canonical signaling kinases (PTEN-PI3K-AKT/PKB and MAPK) and transcription factors (e.g., Myc, p53, NFkB and HNF4A) with a widespread potential to regulate the metabolic phenotype, proliferation and death.

*In silico* analysis of DEGs by IPA and KEGG programs indicated that the synthesis and turnover of phosphatidylinositol bis- and tris-phosphates (IP_2_ and IP_3_) and their receptors played a prominent role in the actions of CBHA and TSA. Our observations corroborate and extend earlier results showing that pan-HDAC inhibitors blunt the PI3K-AKT signaling by at least two different mechanisms [17,19,20]. First, it has been reported that TSA blocked interactions of protein phosphatase-1 with HDACs 1 and 6; this led to increased dephosphorylation of pAkt. Secondly, we have demonstrated that pan-HDACIs CBHA and TSA opposed PI3K-AKT signaling via inducing PTEN gene expression in cardiac myocytes as well in the intact hearts. Based on the network analysis shown here we speculate that PTEN-specific gene networks regulate cell cycle and growth via PLK1, CDC20, MAST1 and LIMK1 kinases.

An extensive review of the literature indicates that HDACIs are capable of blunting the inflammatory response in a number of pathological settings [21]. Apparently, several signaling kinases, including MAPKs, participate in the anti-inflammatory actions of pan-HDACIs. It is significant therefore that both CBHA and TSA inhibited the activation of ERK and TSA inhibited phosphorylation of p38 MAPK in H9c2 cells in a time dependent manner. Earlier observations have also shown that PI3K and MAPK signaling are engaged in extensive crosstalk in the patho-physiology of the heart [22-27]. The activation of ERK via phosphorylation was associated with neoplastic transformation that was inhibited by TSA [28]. Similarly, TSA could also block the activation of ERK signaling induced by TGF-β [19].

We have reported previously that CBHA induced hyper-acetylation of histone H3 (H3-K9) and inhibited its phosphorylation (H3-S10) in IL-18 treated cells [14]. Both CBHA and TSA elicited similar posttranslational modifications of histones in the cardiac chromatin [17]. It has been suggested by Saccani and coauthors that p38 dependent phosphorylation of histone H3 may mark promoters for increased NF-kB recruitment [29]. Based on our limited analysis of changes in the phposphorylatin and acetylation of p65 subunit of NFkB in H9c2 cell treated with CBHA or TSA (Data not shown), we posit that both HDACIs could alter NF-kB recruitment to selected chromatin targets in these cells. These data must be tempered with caution and precise link between NFkB and suppression of anti-inflammatory gene networks by CBHA and TSA remains in the realm of speculation. This is because the regulation of NFkB, consisting of dimeric permutations of c-Rel, RelA, RelB, p50, and p52 subunits, via acetylation is highly complex and context-dependent [30-32].

The cardinal features of maladaptive cardiac hypertrophy include (i) a major shift from fatty acid to glucose oxidation as the main source of fuel [8,33,34], (ii) increased size and contractility of myocytes, and (iii) excessive accumulation of extracellular matrix and fibrosis [35-37]. The induction of TNF-α IFNγ, IL-6, and TGFβ specific gene networks in the cardiac myocytes in response to TSA and CBHA suggests that HDACIs are capable of interfering with cell proliferation (apoptosis and autophagy), pro-inflammatory [38-40] and pro-fibrotic [33,41] mechanisms. Both IPA and KEGG analyses also unraveled a striking effect of HDACIs on the metabolism of lipids, carbohydrates, amino acids, purines and pyrimidines, as well as on the metabolism of glutathione and xenobiotics. The potential reprogramming of gene expression by HDACIs to elicit the gene networks observed here would be expected to alleviate metabolic consequences of pathological cardiac hypertrophy.

Recent observations have demonstrated that pan-HDACIs not only enhance acetylation of histones, but also of numerous other proteins that include transcription factors and enzymes involved in glycolysis, gluconeogenesis and fat and glycogen metabolism [9,42-47]. With regard to the phenotypic changes seen in H9c2 cells treated with CBHA and TSA, it is evident that the signaling cascades induced by both HDACIs culminated in the nucleus to re-program expression of genes that control growth and differentiation (i.e., HDAC7, CBP/p300, NFATc4, MEF-2, myogenin and MyoD) and architecture (i.e., myosin, skeletal muscle actin, tubulin and vimentin) of cardiac myocytes. It was also evident that both CBHA and TSA impinged on a number of common transcription factors Myc, p53, HNF4A and NFkB (predicted by IPA) and E2F, EGR2, AP2, and ETF (predicted by Core_TF), that are known to modulate the expression of genes that regulate S, G and M phases of the cell cycle [48-53]. A role of NFkB in the protection of cardiac myocytes from inflammatory signals, both *in vitro* and *in vivo* is well established; HDACIs are known to regulate NFkB signaling [11,40,54].

We should note that *in silico* predictions of the IPA and CORE_TF programs with respect to the putative transcription factors are limited in two ways. First, these analyses only provide a snapshot of transcription at 6h and 24h and need to be extended on both sides of the timescale used here. Second, the exact dynamics of induction of various TFs need to be experimentally validated. With these caveats notwithstanding, it is noteworthy that the preponderance of the TFs involved in the regulation of gene expression in response to TSA or CBHA were not identical. Thus, the IPA predicted HNF4A, Myc, p53 and NFkB to be the dominant transcription factors; in contrast, the Core_TF program predicted the preponderance of E2F1, AP2, EGR2, ETF, Sp1 and KROX. These apparently dissimilar predictions of TFBS that mediate epigenetic regulation of DEGs likely reflect the uniqueness of the two programs. The IPA assigns nodes in gene network using focus molecules and their known relationships based on published observations stored in the Ingenuity Pathways Knowledge Base. In contrast CORE_TF program uses the focus genes exclusively and directly interrogates their promoters for TFBS. Nevertheless, both IPA and Core_TF programs give complementary information on the common biological processes (e. g., proliferation, cell cycle and apoptosis) by pan-HDAC inhibitors. The known regulatory interrelationships among the dominant TFs predicted by IPA and Core_TF support this notion. For instance, NFkB is known to interact with the regulatory regions of Myc and cyclin D1, both critical components of cell cycle regulation. Similarly, Myc regulates the expression of E2F via cyclin D1. A differential expression of p53 and CDKNA predicted by IPA is highly significant. The regulation of p53 expression is mechanistically linked to E2F (a key transcription factor predicted by Core_TF), CDKs and cyclins. The p53 also forms a prominent network that directly connects it to p21 and cyclin D1 both of which are involved in the regulation of E2F, NF-Y and ETF transcription factors. Finally, it should be noted that cyclins, CCNA2, CDC2 and herpud1 are *bona fide* targets of NF-Y regulation.

## Conclusions

Based on these data we conclude that pan-HDAC inhibitors impinge on a number of key regulatory gene networks to profoundly alter the phenotype of H9c2 cardiac myocytes to facilitate their survival in the face of potential inflammatory pathways evoked by pro hypertrophy agents (e.g., phenylephrine, Ang-II and IL-18). The cytokine-specific gene networks, signaling pathways and transcription factors putatively perturbed by pan-HDAC inhibitors reported here provide a potential platform to test a number of hypotheses related to the known specificity and toxicity of pan-HDAC inhibitors *in vitro* and *in vivo*[12,18,39].

## Methods

### Cell culture

H9c2 cells were purchased from American Type Culture Collection, Bethesda MD, were grown in Dulbecco’s minimum essential medium (DMEM) containing 10% fetal bovine serum (Hyclone, Logan, UT), 2 mM glutamine and 1% Penicillin/Streptomycin. Cells were allowed to reach about 80% confluence in complete culture medium. The cultures were incubated for additional 24h in serum-free medium prior to experimental treatments, as outlined previously [14]. Six replicate cultures of H9c2 cells each were treated with either CBHA (1 μM) or TSA (100 nM); aliquots of parallel cultures incubated in complete growth medium for 6h and 24h served as control for gene expression analysis.

### Gene expression profiling

RNA was extracted from H9c2 cells by the Trizol method followed by a cleaning up of RNA samples with an RNeasy clean up kit (Qiagen Inc., Valencia, CA). The total yield and quality of RNAs were established by measuring absorbance at 260nm/280nm in a spectrophotometer and size-fractionation by electrophoresis in 1% agarose gels, respectively. Two hundred ng aliquots of total RNA per sample were used for cDNA and cRNA synthesis; we used Illumina® TotalPrep™ RNA Amplification Kit (Applied Biosystems/Ambion, Austin, TX). Aliquots of amplified and labeled cRNA (750–1500 ng) were hybridized to Illumina RatRef-12 Expression BeadChips containing >22,000 transcripts (Illumina Inc., San Diego, CA). After washing and staining, chips were scanned on the Illumina 500GX BeadArray Reader using Illumina BeadScan image data acquisition software. The data acquisition, processing and normalization of the microarray data were done with Illumina GenomeStudio software (version 1.5.10) to generate an output file for statistical analysis.

### Statistical analyses of differential gene expression

Statistical, mulitvariate and clustering analyses were performed in GeneMaths XT (Applied Maths, Belgium). The identification of differentially expressed genes was based on (1) Illumina detection values ≥ 0.99 for all samples in at least one experimental or control group and (2) ANOVA p-value ≤0.01; 3), absolute fold change ≥2.0 and independent t-test p-value ≤ 0.01 for any experimental group versus its respective control group. Principal component analysis (PCA) was performed using signal values for probe sets with detection values ≥0.99 for all samples in at least one experimental or control group; signal values were log2 transformed and standardized by row mean centering prior to PCA. Unsupervised hierarchical clustering of DEGs was performed using UPGMA method (Un-weighted Pair Group Method using Arithmetic averages) that uses Euclidean distance as the similarity metric. Sample clustering was done using Complete Linkage method with Pearson correlation as the similarity metric. Venn diagrams were generated by Boolean intersection of gene IDs for DEGs from the indicated pair-wise comparisons.

### Bioinformatics analyses

Gene annotation and Gene Ontology (GO) were obtained from the National Center for Biotechnology Information (http://www.ncbi.nlm.nih.gov) and the Gene Ontology Consortium (http://amigo.geneontology.org).Analyses of GO enrichment and KEGG (Kyoto Encyclopedia of Genes and Genomes; http://www.genome.jp/kegg) biochemical pathways were performed using WebGestalt (http://bioinfo.vanderbilt.edu/webgestalt). Hypergeometric test p-values were used to estimate the significance of enrichment of specific GO categories or pathways.To search for over-represented transcription factor binding sites (TFBS) in the DEGs induced by HDACIs, we used a web-based program CORE_TF (55) (http://www.LGTC.nl/CORE_TF). This program was used to search for common TF binding motifs, derived from postion based matrices from the TRANSFAC^R^ database. The search for TFBS was restricted to the 1000 bases upstream of the transcription start site (TSS). The output p-values and promoter hits were obtained after correcting for a false discovery rate of 1%. The methods have been detailed previously [55].

### Ingenuity pathways analysis

The canonical network models of DEGs were developed using the IPA (version 8.7) (http://www.ingenuity.com) as outlined in detail previously [17]. The Illumina gene lists were uploaded as a text file and each gene identifier was mapped to its corresponding gene object. An initial gene set of DEGs was first overlaid onto the set of all catalogued interactions and focus genes contained in the IPA library of canonical pathways. To start building networks, the application queries the Ingenuity Pathways Knowledge Base for interactions between Focus Genes and all other gene objects stored in the knowledge base and generates a set of networks each with no more than 35 genes/proteins. The IPA then computes a score for each network according to the fit of the user’s set of significant genes. The score is derived from a *p* value that denotes the likelihood of a Focus Gene’s presence in a network due to chance. The networks graphically denote nodes and edges, or lines (the biological relationships between the nodes). Assignment of nodes in gene network is made using published observations stored in the Ingenuity Pathways Knowledge Base. A Fischer’s exact test was used to calculate a *p* value predicting the probability that the biological function assigned to that network is explained by chance alone.

### PCR-based quantification of gene expression

RNA was extracted from control or treated H9c2 cardiac myocytes using TRIzol RNA extraction reagent (Invitrogen Life Technologies). Total RNA was precipitated with ethanol, concentrated by centrifugation and dissolved in diethylpyrocarbonate (DEPC)-treated water. Aliquots of 800 ng of RNA were used to synthesize cDNA (SuperScript III, Invitrogen). Gene-specific primers and TaqMan probes for quantitative RT-PCR (Q-PCR) were designed using Universal Probe Library (Roche) as detailed previously [17]. The Cp values for each HDAC and Sirtuin gene were normalized to the Cq values of the constitutively expressed ß-actin gene.

### Western blot analysis

Total proteins from H9c2 cells were extracted using radio-immunoprecipitation (RIPA) buffer according to the manufacturer’s protocol (Santa Cruz Biotechnology, CA). The nuclear and cytoplasmic and fractions were separated using the NE-PER^TM^ (http://www.thermo.com/pierce) method [56]. For western blot analysis, equal amounts of protein from each sample were separated using 10% SDS-PAGE. After electrophoresis, the protein samples were transferred to Immobilon-P membranes (Millipore Corp., Bedford, MA) using a Trans-Blot electrophoresis transfer cell (Bio-Rad Laboratories, Inc., Hercules, CA). Various HDACs, sirtuins and MAP kinases were detected on western blots with mono-specific primary antibodies (Santa Cruz). Anti-ERK, anti-phospho-ERK or anti-phospho-p38 antibodies were obtained from Cell Signaling Technology (Beverly, MA). The blots were sequentially reacted with primary antibodies followed by horseradish peroxidase-conjugated anti-rabbit IgG antibodies according to manufacturer’s instructions (Santa Cruz). Chemi-luminescence signals developed using ECL Plus kit (Amersham-Pharmacia Biotech, Piscataway, NJ). Some blots were stripped and re-probed with anti-ERK or p38 antibodies to determine equivalency of protein loading. The data from 3–4 replicate experiments were quantified by densitometry, normalized against total ERK or p38 or actin, and subjected to statistical analysis, as outlined previously [14,57].

## Competing interests

The authors declare that they have no competing interests.

## Authors’ contributions

RR and GM Conceived and designed most of the experiments. GM, PA and NB performed wet laboratory experiments; NB and HC carried out in silico analysis of transcription factor binding sites. RR and GM wrote the paper. All authors read and approved the final manuscript.

## Supplementary Material

Additional file 1 **Figure S1.** Major gene networks formed by DEGs in Clusters A-F in H9c2 cells treated with TSA for 6 h. Cluster A formed gene network with INF γ TP53andAkt as central nodes connected by direct (solid lines) or indirect (broken lines) to genes that were either up regulated (pink and red) or down regulated (green). Cluster C showed the intracellular gene networks with TNFα, MYC and TP53 and the signaling molecules ERK-JNK-p38MAPK and NFκB forming the central nodes. Cluster D had dominant gene network specifying TP53 and YY1 as the central nodes. Clusters F demonstrated TNFα and IL-6 as major nodes connected to mostly down regulated genes (green). Click here fof file

Additional file 2 **Figure S2.** IPA of DEGs in Clusters A-F induced by TSA in H9c2 cells at 24 h. Cluster A demonstrated the intracellular gene network with TNF-α, MYC, FOS, HNF-4A and TP53 and the signaling molecules PI3K-Akt-ERK-JNK-p38MAPK and NFκB forming the central nodes. Cluster C demonstrated the intracellular gene networks with TNF-α, TP53, MYC and HNF-4A and the signaling molecules PI3K-Akt-ERK-JNK-p38MAPK and NFκB as the central nodes. Cluster D demonstrated TGFβ, TP53 and CDKN1A genes as the central nodes in the gene network. Cluster F demonstrated TNF-α and INFγ connected with the signaling nodes of PI3K-AKT- ERK-JNK-p38MAPK and NFκB. Click here fof file

Additional file 3 **Figure S3.** Intracellular gene networks formed by DEGs in Clusters A-F in H9c2 cells in response to CBHA treatment for 6 h. Cluster A formed dominant gene networks with TNF-α INFγ and TP53 as the central nodes connected directly (solid lines) or indirectly (broken lines) to genes that were either up regulated (pink and red) or down regulated (green). Cluster B depicted TNFα, INΦγ, TP53 and MYC as the central nodes in the gene network. Cluster D demonstrated TP53 and CDKN1A genes as the central nodes in the gene network. Cluster E displayed the dominant nodes, centered by TNF−α TP53, CDKN1A and INFγ. Click here fof file

Additional file 4 **Figure S4.** Major gene networks formed by DEGs in Cluster A-F in H9c2 cells in response to CBHA treatments for 24 h. Cluster A formed gene networks with TNF−α and the signaling nodes PI3K-AKT-JNK and NFκB. Cluster B depicted TNF-α, INFγ, TP53, MYC and CDKN2A and the signaling molecules PI3K-Akt-ERK-JNK-p38MAPK and NFκB as the central nodes in the gene network. Cluster D demonstrated TP53, YY1 and CDKN1A genes as the central nodes in the gene network. Cluster E displayed the dominant nodes, centered by TNF-α, ΙNFγ TP53 and CDKN1A. Click here fof file
