# Supplementary figures and images for: Pan-histone deacetylase inhibitors regulate signaling pathways involved in proliferative and pro-inflammatory mechanisms in H9c2 cells
Source: BMC Genomics. 2012 Dec 18;13:709. doi: 10.1186/1471-2164-13-709 (PMC3561284; doi:10.1186/1471-2164-13-709)

## Slide 1
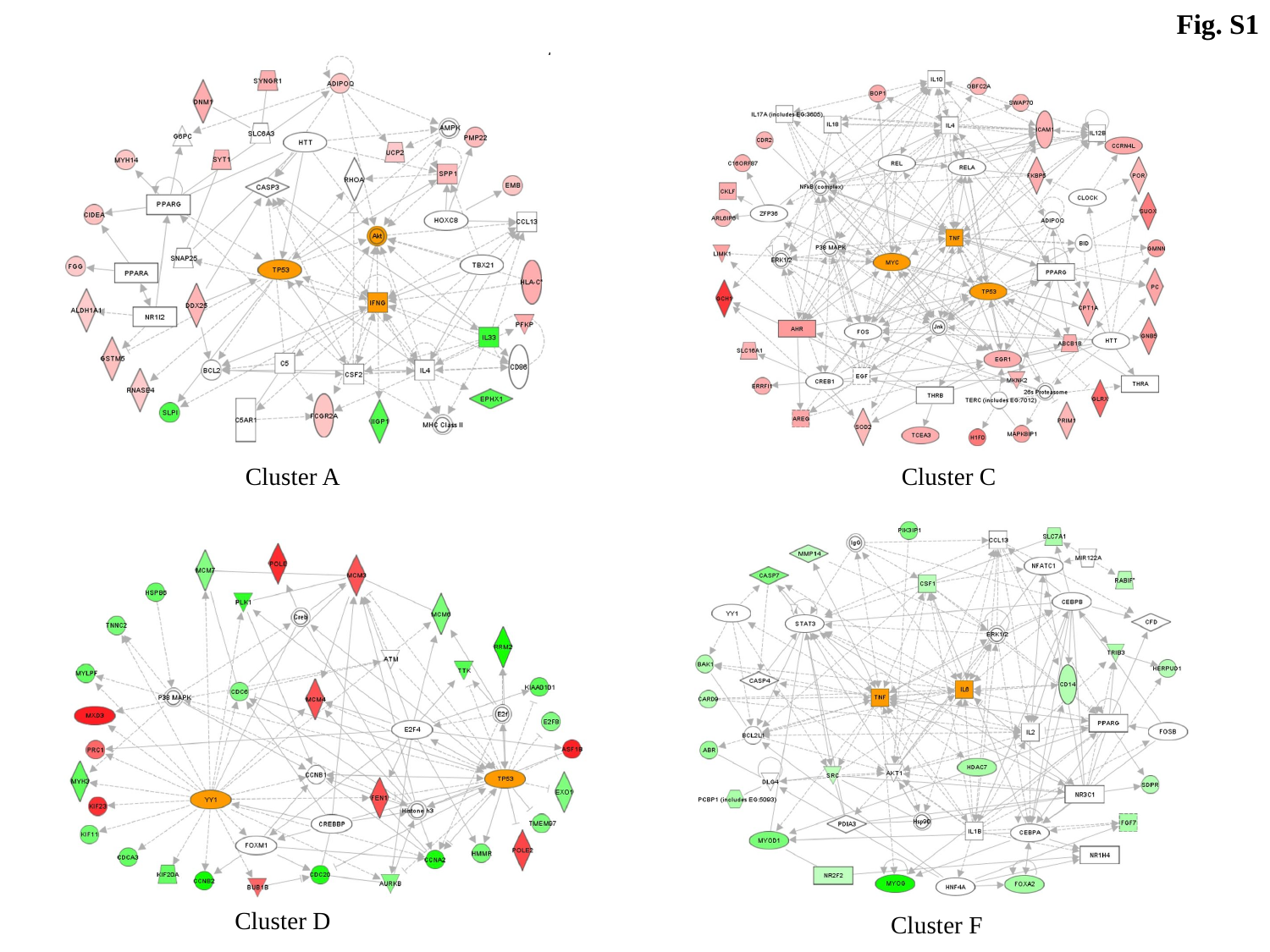

Fig. S1
Cluster A
Cluster C
Cluster D
Cluster F

Supplement: Additional file 1 — Figure S1. Major gene networks formed by DEGs in Clusters A-F in H9c2 cells treated with TSA for 6 h. Cluster A formed gene network with INF γ TP53andAkt as central nodes connected by direct (solid lines) or indirect (broken lines) to genes that were either up regulated (pink and red) or down regulated (green). Cluster C showed the intracellular gene networks with TNFα, MYC and TP53 and the signaling molecules ERK-JNK-p38MAPK and NFκB forming the central nodes. Cluster D had dominant gene network specifying TP53 and YY1 as the central nodes. Clusters F demonstrated TNFα and IL-6 as major nodes connected to mostly down regulated genes (green). [file 1471-2164-13-709-S1.pptx]

## Slide 1
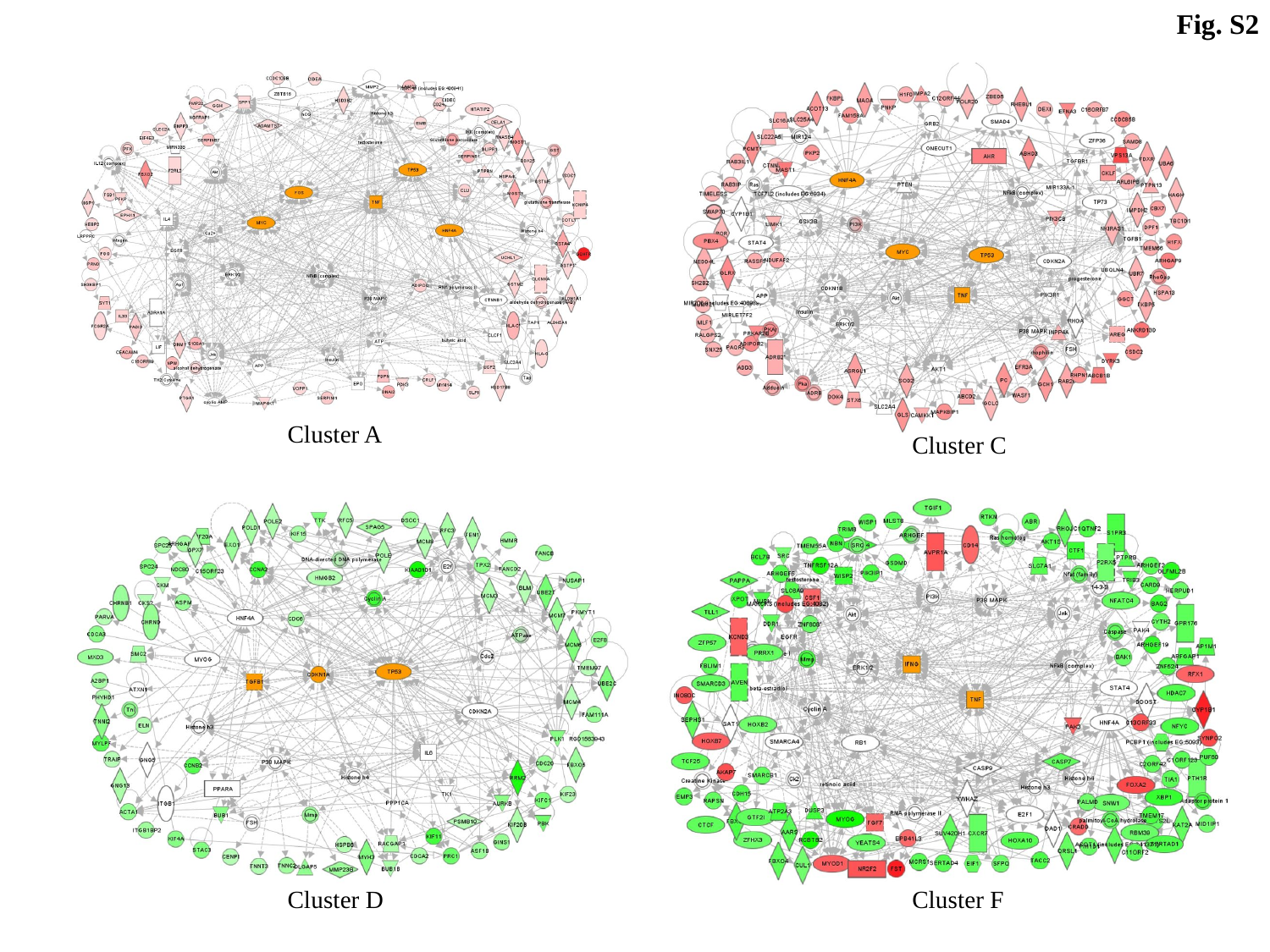

Fig. S2
Cluster A
Cluster C
Cluster D
Cluster F

Supplement: Additional file 2 — Figure S2. IPA of DEGs in Clusters A-F induced by TSA in H9c2 cells at 24 h. Cluster A demonstrated the intracellular gene network with TNF-α, MYC, FOS, HNF-4A and TP53 and the signaling molecules PI3K-Akt-ERK-JNK-p38MAPK and NFκB forming the central nodes. Cluster C demonstrated the intracellular gene networks with TNF-α, TP53, MYC and HNF-4A and the signaling molecules PI3K-Akt-ERK-JNK-p38MAPK and NFκB as the central nodes. Cluster D demonstrated TGFβ, TP53 and CDKN1A genes as the central nodes in the gene network. Cluster F demonstrated TNF-α and INFγ connected with the signaling nodes of PI3K-AKT- ERK-JNK-p38MAPK and NFκB. [file 1471-2164-13-709-S2.pptx]

## Slide 1
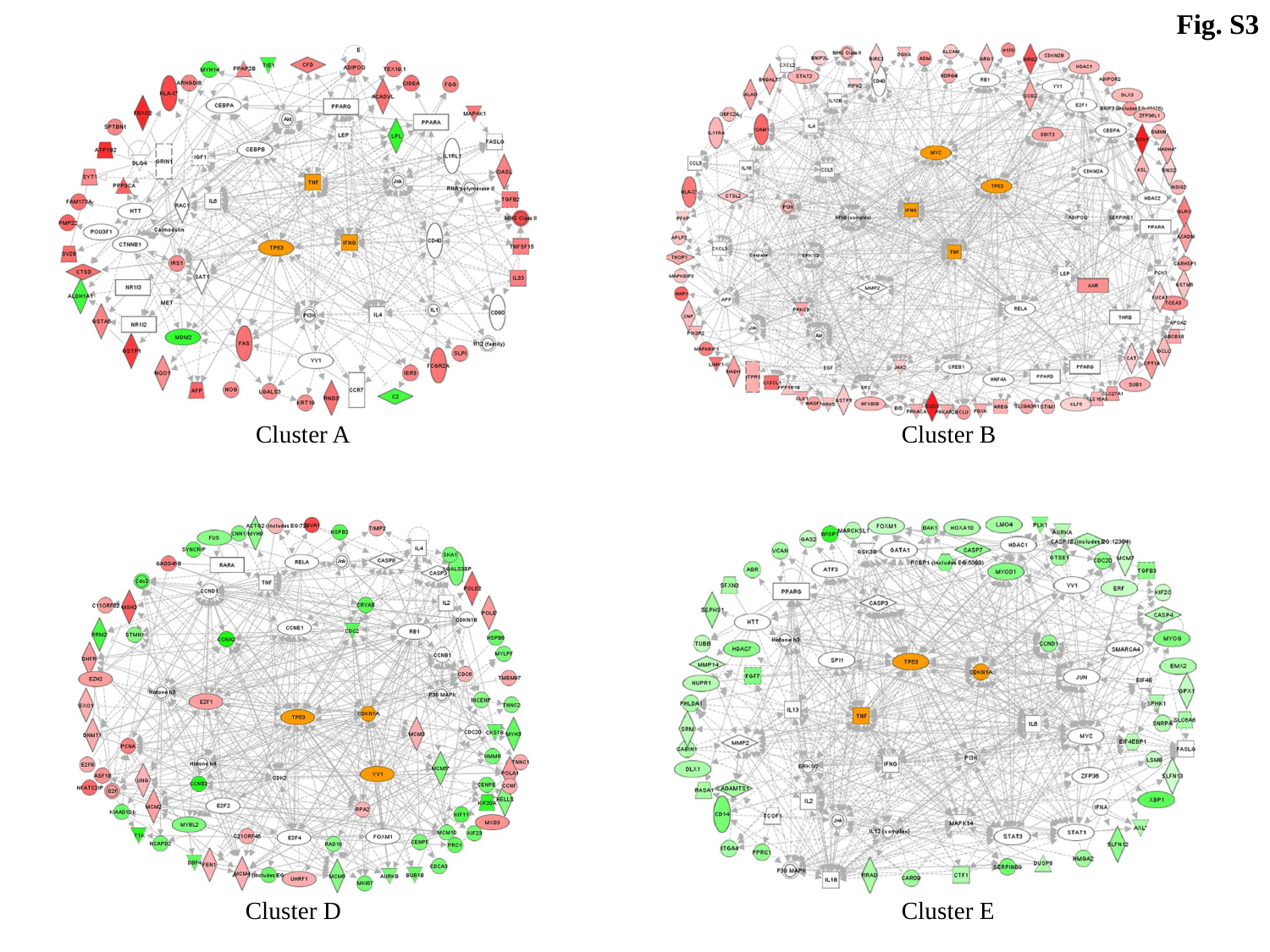

Fig. S3
Cluster A
Cluster B
Cluster D
Cluster E

Supplement: Additional file 3 — Figure S3. Intracellular gene networks formed by DEGs in Clusters A-F in H9c2 cells in response to CBHA treatment for 6 h. Cluster A formed dominant gene networks with TNF-α INFγ and TP53 as the central nodes connected directly (solid lines) or indirectly (broken lines) to genes that were either up regulated (pink and red) or down regulated (green). Cluster B depicted TNFα, INΦγ, TP53 and MYC as the central nodes in the gene network. Cluster D demonstrated TP53 and CDKN1A genes as the central nodes in the gene network. Cluster E displayed the dominant nodes, centered by TNF−α TP53, CDKN1A and INFγ. [file 1471-2164-13-709-S3.pptx]

## Slide 1
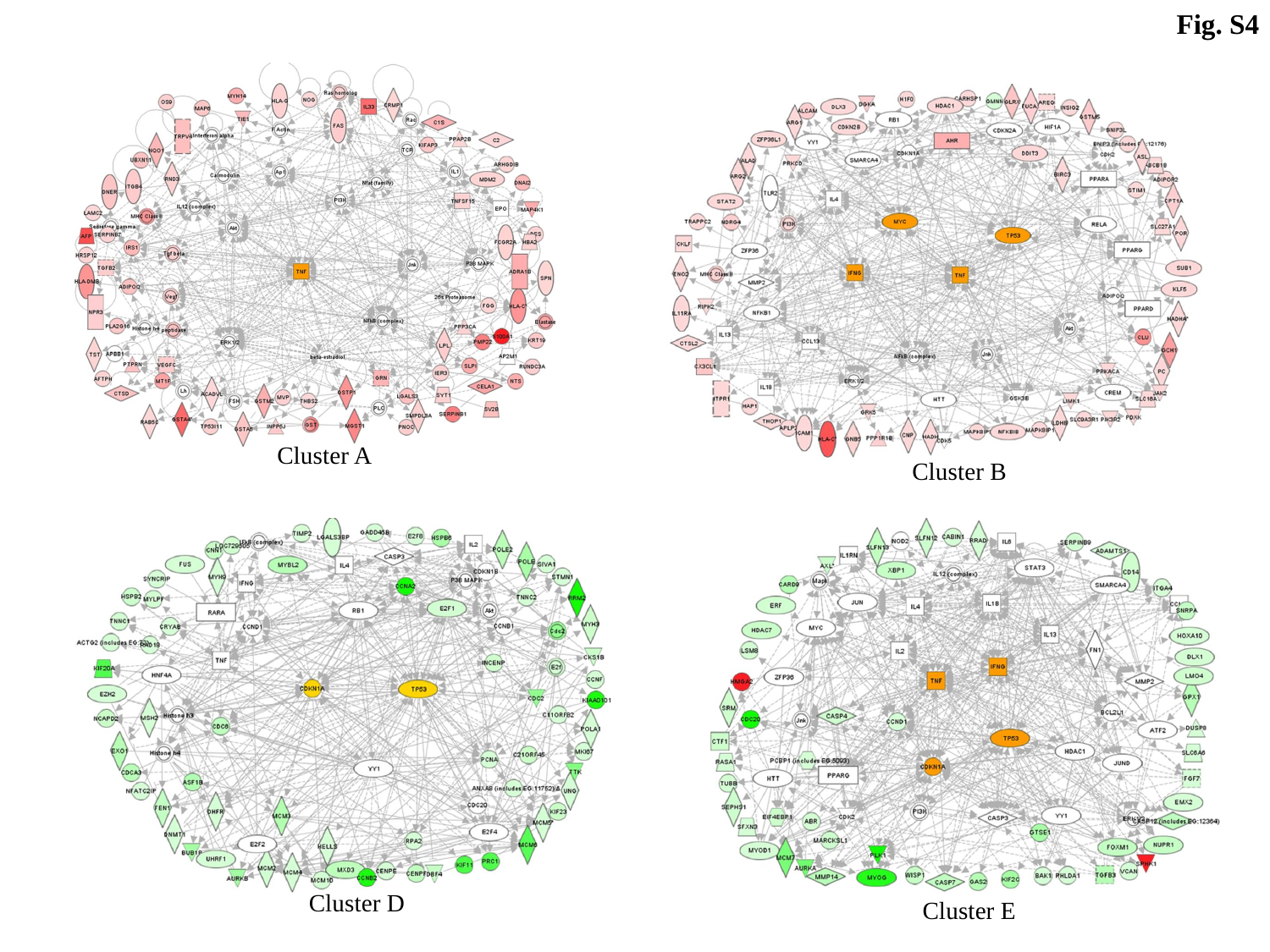

Fig. S4
Cluster A
Cluster B
Cluster D
Cluster E

Supplement: Additional file 4 — Figure S4. Major gene networks formed by DEGs in Cluster A-F in H9c2 cells in response to CBHA treatments for 24 h. Cluster A formed gene networks with TNF−α and the signaling nodes PI3K-AKT-JNK and NFκB. Cluster B depicted TNF-α, INFγ, TP53, MYC and CDKN2A and the signaling molecules PI3K-Akt-ERK-JNK-p38MAPK and NFκB as the central nodes in the gene network. Cluster D demonstrated TP53, YY1 and CDKN1A genes as the central nodes in the gene network. Cluster E displayed the dominant nodes, centered by TNF-α, ΙNFγ TP53 and CDKN1A. [file 1471-2164-13-709-S4.pptx]
